# Adsorption of Cu (II) Ions Present in the Distilled Beverage (Sugar Cane Spirit) Using Chitosan Derived from the Shrimp Shell

**DOI:** 10.3390/polym14030573

**Published:** 2022-01-31

**Authors:** Lucely Nogueira dos Santos, Alberdan Silva Santos, Kelly das Graças Fernandes Dantas, Nelson Rosa Ferreira

**Affiliations:** 1 Graduate Program in Food Science and Technology, Federal University of Pará, Belém 66077-000, PA, Brazil; lucelynogueira@gmail.com; 2 Faculty of Chemistry, Institute of Exact and Natural Sciences, Federal University of Pará, Belém 66075-110, PA, Brazil; alberdan.ufpa@gmail.com (A.S.S.); kdgfernandes@gmail.com (K.d.G.F.D.); 3 Faculty of Food Engineering, Technology Institute, Federal University of Pará, Belém 66077-000, PA, Brazil

**Keywords:** adsorption, sugar cane spirits, copper (II), removal of potentially toxic element, chitosan

## Abstract

Cachaça (sugar cane spirit) is a typically Brazilian distilled beverage. Copper ions can be present in craft beverages despite their acceptance in the national and international market. This study aims to evaluate the efficiency of chitosan as an adsorbent in removing copper (II) from cachaça. The structural characteristics of the obtained chitosan and the effect of adsorbed copper were evaluated by Fourier Transform Infrared Spectroscopy (ATR-FTIR), viscosimetry, X-ray diffraction (XRD), and scanning electron microscopy (SEM). The deacetylation reaction from chitin (shrimp shell) resulted in chitosan with a deacetylation degree of 88.9% (potentiometric titration) and 86.9% (FTIR), low crystallinity, and an estimated molecular weight of 162.96 kDa. The copper reduction rate was 84.09% evaluated by spectrophotometric titration and microwave-induced plasma optical emission spectrometry (MIP–OES). The amine groups of chitosan had adsorption affinity with copper ions, and the kinetic analysis showed a better fit of the data by the Elovich equation, suggesting that the chemosorption mechanism controlled the kinetic process. The results suggest that chitosan has the potential to improve the quality and safety of cachaça.

## 1. Introduction

In the production of cachaças, copper has the essential function of catalyzing chemical reactions and contributing to the reduction of acidity and levels of aldehydes formed during the drink’s preparation stages [1,2]. The presence of copper comes from the stage of distillation in alembic pots. This metal can be observed in the alcoholic distillate due to a natural drag of the copper during the distillation. However, ineffective internal cleaning can increase concentration levels [3]. Copper ions above the specified limits can characterize flaws in the process and cause damage to human health and the environment [2]. Brazilian legislation establishes a limit of 5 mg mL^−1^ of copper II in cachaça [4]. However, the legislation of other countries does not allow more than 2 mg mL^−1^ of copper (II) in alcoholic distillates.

Several techniques have been developed to remove potentially toxic elements, mainly in aqueous solutions. These techniques include chemical oxidation, adsorption, ion exchange, and membrane separation [5]. The adsorption technique is considered simple, economical, efficient, and is a good proposal for removing metal ions [6,7]. The adsorptive capacity of various materials has been tested to reduce copper ions in cachaça, such as filters with adsorbents, activated carbon, silica, resins, and clays [8]. However, some of these materials are costly and can remove, in addition to copper, substances essential for the flavor and aroma of the beverage, affecting its chemical quality. To reduce this effect, it is possible to use natural or modified biopolymers, such as chitosan [9,10].

Chitosan is a natural biopolymer (derived from chitin *N*-deacetylation), formed by β-1,4 d-glucosamine units linked to *N*-acetylglucosamine residues. Chitosan has two hydroxyl groups and a free amine group in its glycosidic residues. Free amine groups act as active sites with high adsorption capacity [11,12]. The increasing use of natural adsorbents to remove potentially toxic elements, phenols, paints, and other pollutants [13] is due to the chemical characteristics of the functional groups in their structure. Thus, chitosan is an important bio adsorbent [14,15].

Shrimp shells can be used as a precursor to chitosan, as a large-scale by-product of the fishing industry, since they present good economic viability for use at industrial scale. Shrimp shells consist of a network of various components such as proteins, carbonates, pigments, and chitin [16]. Several types of research have already been carried out on the use of this waste. These studies include isolation and characterization of carotenoproteins [17], extraction of shrimp oil rich in omega-3, and astaxanthin [18], and use in the preparation of animal feed [19].

Although the traditional deacetylation procedure, with its energy liability and chemical waste, is still widely used, the search for clean technologies has been reported. Clean technologies should consider reducing energy consumption and reducing the amount of waste generated [20]. In this study, microwave technology was an alternative for the deacetylation of chitin. This technology reduces chemical products, lowers energy consumption, and shorter processing time [21].

The present study uses chitin from the deacetylated shrimp shell (chitosan) to reduce the Cu (II) content in cachaça from copper alembic stills. It shows the capacity of chitosan as Cu (II) adsorbent under experimental conditions. The results suggest that chitosan without chemical modification (original chitosan) can remove Cu ions in beverages such as cachaça, opening up possibilities for applications of this biopolymer to improve consumer safety without affecting the sensory quality of the distilled beverage. 

## 2. Materials and Methods

### 2.1. The Samples, Reagents and Solutions

A local fishing company (Amazonas Industrias Alimentícias S/A Amasa, Belém, Pará, Brazil) supplied the waste from shrimp processing (exoskeleton). The cachaça used in this study was purchased from a distillery Cachaça from Amazônia in (Pará, Brazil). CuSO_4_·5H_2_O was used to prepare a standard curve. All solutions were prepared with analytical grade reagents and high purity distilled water.

### 2.2. Chitosan Preparation

#### 2.2.1. Precipitation of Chitin

The residues (exoskeleton) were dried in an oven with air circulation (Tecnal- model TE-394/3) at 60 ± 1 °C for 6 h, crushed in a mill (Labor Muszeripari Muvek-Esztergom-Type: OB-136) and sieved. Fine particles with a diameter of 351 µm were obtained. Chitin extraction involved the stages of demineralization and deproteination, according to the method proposed with modifications in [22]. The demineralization step was carried out in three immersion cycles (two cycles of 15 min and one cycle of 60 min) in hydrochloric acid solution (0.55 M), in the solid/solution ratio of 10/100 (*w*/*v*). The temperature was maintained at 25 ± 1 °C with a constant stirring agitator plate (Brand: Fisatom model: 752A). The contents were filtered and washed with water until pH ~7.0. The demineralized material was subjected to three cycles of alkaline immersions for 20 min with sodium hydroxide (Neon) (0.3 M). The solid/solution ratio was 10/100 (*w*/*v*) at a temperature of 83.0 °C ± 2.0 °C, under constant agitation, for protein removal. The material was washed with water until pH ~7.0. It was then filtered and dried at 60 ± 1 °C for 2 h. The product obtained was called shrimp chitin (*Q_i_*) and was a light brown powder.

#### 2.2.2. Deacetylation of Chitin

The deacetylation step was performed using microwave irradiation according to the method of Sagheer and collaborators [23]. Chitin was immersed in NaOH solution 50% (*w*/*v*), in a chitin/solution ratio of 1/100 (*w*/*v*) for 24 h, at an ambient temperature of 25 °C. Then, the entire contents (100 mL) were placed in 250 mL porcelain crucibles and placed in the microwave oven (Brastemp model: Single) with power adjusted to 700 W for 10 min. After cooling, the sample was washed with distilled water until pH ~7.0, vacuum filtered and oven-dried at 60 ± 1.0 °C for 2 h. The product of this process was called chitosan (*Q_t_*).

### 2.3. Characterization of Chitosan

The degree of deacetylation (DD) was evaluated using the linear potentiometric titration method [24] and confirmed by attenuated total reflectance Fourier Transform Infrared Spectroscopy (ATR-FTIR) [25].

#### 2.3.1. Potentiometric Titration and ATR-FTIR Analysis

Potentiometric titration was performed using the linear method [24]. 0.25 g of chitosan was dissolved in 20 mL of 0.1 N HCl solution, made up to 100 mL with distilled water and titrated with NaOH (0.1 N), in the range of pH 2.0 to 6.0 (range of non-protonation of chitosan). A portable digital pH meter (Lab1000, model Mpa 210-MS Tecnopon, Piracicaba, São Paulo, Brazil) was used to measure the pH. The system was kept under constant agitation in a thermostatically controlled system at 25 ± 0.5 °C. The linear titration curve was obtained by plotting f(x) as a function of the NaOH volume. At the end of the titration, the volume of NaOH (Ve) was estimated by extrapolating the linear titration curve to f(x) = 0.

The value of f(x) corresponding to the volume of NaOH added was calculated using Equation (1):

(1) $$f\left(x\right){=(V}_{0}+V/N_{B})\cdot \left(\left[H^{+}\right]-\left[{\mathrm{OH}}^{-}\right]\right)$$

where V_0_ is the initial volume of chitosan solution (mL); V is the volume of NaOH used in the titration (mL); N_B_ is the molar concentration of NaOH (mol L^−1^); [H^+^] is the concentration of H^+^ (mol L^−1^), and [${\mathrm{OH}}^{-}$] is the concentration of ${\mathrm{OH}}^{-}$ (mol L^−1^).

The DD was calculated using Equations (2) and (3):

(2) $$\mathrm{DD}\left(\%\right)=Ø/\left[\left(W-161Ø\right)/204+Ø\right]\times 10$$

(3) $$Ø=\left(N_{A}V_{A}{-N}_{B}V_{e}\right)/1000$$

where N_A_ is the concentration of HCl (mol L^−1^); V_A_ is the volume of HCl (mL); N_B_ is the concentration of NaOH (mol L^−1^); V_e_ is the volume of NaOH at the end of the titration (mL); W is the mass of the sample (g); 161 corresponds to the molar mass of the glycosidic unit in g mol^−1^, 204 corresponds the molar mass of chitin (mg mol^−1^ per unit).

Attenuated Total Reflectance Fourier Transform Infrared Spectroscopy (*ATR-FTIR*) was used to identify the main functional groups and the structural changes in chitin/chitosan. The analysis was carried out between 4000 cm^−1^ to 600 cm^−1^ with a resolution of 8 cm^−1^ and 32 scans. The equipment used was Cary 360 (Agilent, Santa Clara, CA, USA) with zinc selenide crystal (ZnSe) and an ART module.

The spectra were edited and analyzed using the Spectragryph^®^ software (version 1.2.14/2020/ Alemanha, Oberstdorf, Germany). The baselines of the spectra were adjusted using the Advanced Baseline function. The areas corresponding to the functional groups of amine (1310 cm^−1^) and CH_2_ (1420 cm^−1^) of chitosan were calculated using the Integration by Area function. The DD was calculated by integrating the areas of the specific bands [25] according to Equations (4) and (5).

(4) $$A_{1310}/A_{1420}=0.3822+0.0313\;\mathrm{DA}$$

(5) Degree of Deacetylation (%DD)=100−DA

where DA is the Degree of Acetylation; A_1310_ is the area under the curve of the infrared spectrum band with a wavenumber of 1310 cm^−1^; A_1420_ is the area under the curve of the infrared spectrum band a wavenumber of 1420 cm^−1^.

#### 2.3.2. Determination of the Molecular Weight of Chitosan by Viscosimetry

The *Q_t_* was dissolved in sodium acetate buffer (0.2 M) and acetic acid (0.1 M). Viscosity measurements were performed on a Cannon-Fenske viscometer (Schott AVS 350, Schott-instruments, Mainz, Germany) at 25 ± 1.0 °C. From the kinematic viscosity (v) (cm^2^ s^−1^) of the solutions, the specific viscosity (η_sp_) was calculated. The intrinsic viscosity [η] was obtained by extrapolating the viscosity data to infinite dilution, according to the Huggins [26], according to Equation (6).

(6) $$\eta_{\mathrm{sp}}/C=\left[\eta \right]+K_{H}{\left[\eta \right]}^{2}\cdot C$$

where η_sp_ is the specific viscosity; ${(\eta }_{\mathrm{sp}}/C)$ (mL g^−1^) is the reduced viscosity; C is the concentration of polymer (g mL^−1^); [η] is the intrinsic viscosity (mL g^−1^), and K_H_ is the Huggins constant.

The molecular weight of *Q_t_* was determined from the value of the intrinsic viscosity [η] using Equation (7) (Mark–Houwink-–Sakurada), which relates intrinsic viscosity to the molecular weight [27].

(7) $$\left[\eta \right]={K^{\prime }M}_{V}{}^{\alpha }$$

where [η] is the estimate of intrinsic viscosity; K′ and α are constants for a given solvent and temperature (K′ = 0.074 and α = 0.76) [27], and M_V_ is the viscosimetric molar mass.

### 2.4. Morphological Analysis of Chitosan

#### 2.4.1. Scanning Electron Microscopy

The morphology of the *Q_i_*, *Q_t_* and chitosan samples adsorbed with copper (*Q_c_*) were analyzed by scanning electron microscopy (SEM) Hitachi electron microscope model TM 3000 (Tokyo, Japan). EDS dispersive energy spectroscopy, used to identify the elemental composition of the materials, was coupled to the SEM. Double-sided carbon adhesive tape fixed the samples, and the area reading was performed with a voltage acceleration of 15 kV with an acquisition time of 100 s.

#### 2.4.2. X-ray Diffraction (XRD)

The XRD analyzes were performed in PANalytical Empyrean model (PANalytical, Malvern, UK) equipped with ceramic X-ray tubes (Co anode) (Kα1 = 1.789010 Å), Kβ Fe filter, PIXCEL^3D^-Medpix^3^ 1 × 1 detector with a voltage of 40 kV, current of 35 mA, step size 0.0263° in 2θ, scanning from 3.00° to 95.00° in 2θ, time/step of 59.92 s. Data acquisition was made with the X’Pert Data Collector software, version 5.1, and the data treatment with the X’Pert HighScore Plus software, version 4.7 (Malvern Panalytical, Malvern, UK). 

The comparison of the diffractogram with the standards of the ICDD-PDF (International Center for Diffraction Data—Powder Diffraction File) database helped identify *Q_i_* and *Q_t_*. The crystallinity index was calculated [28] using the following Equation (8).

(8) $$I_{\mathrm{CR}}{=[I}_{\max}{-I}_{0}/I_{\max}]\times 100$$

where I_CR_ is the crystallinity index; I_max_ is maximum intensity, and I_0_ is the initial intensity base of the values of the 2θ positions of the characteristic peak curve.

### 2.5. Copper Content in Cachaça

#### 2.5.1. Spectrophotometric Titration with EDTA

The efficiency of copper ion removal in cachaça was calculated based on the analysis of the total ion concentration performed by spectrophotometric titration with EDTA (ethylenediaminetetraacetic acid, disodium salt) (Nuclear). The reading was carried out at a wavelength of 745 nm (visible region) using spectrophotometer-UV-Visible (Thermo; model: Genesys) [2]. The copper content was determined by comparing the absorbance values in the cachaça sample with the absorbance values for a previously constructed analytical curve. The limits of copper quantification were established between 10 to 200 mg L^−1^ using CuSO_4_·5H_2_O as standard.

#### 2.5.2. Microwave-Induced Plasma Optical Emission Spectrometry (MIP OES)

The quantification of copper was performed using a microwave-induced plasma optical emission spectrometer MIP OES (model 4100, Agilent Technologies Melbourne, Melbourne, Australia). The copper content was evaluated in the samples of cachaça (Pará-Brazil), cachaça doped with copper (50 mg L^−1^) and cachaça after the adsorption process (under equilibrium condition). The solutions were prepared with ultrapure water obtained from a Sinergy-UV purification system (Millipore, Bedford, MA, USA) with resistivity 18.2 MΩ cm. The analytical curve and samples were acidified with 65% *v*/*v* nitric acid (Sigma-Aldrich, Darmstadt, Alemanha, Germany) previously purified. The limit of quantification was 0.006 mg L^−1^.

### 2.6. Evaluation of Cu (II) Adsorption in the Chitosan Matrix

The effect of the contact time between adsorbent and adsorbate was evaluated. A solution with copper at a concentration of 50 mg L^−1^ was prepared from Cu (II) sulfate pentahydrate (CuSO_4_·5H_2_O), using the pot still cachaça as a solvent. Different masses of *Q_t_* (6, 4 and 2 mg mL^−1^) were added in 30 mL of the cachaça solution with pH 4.0. Each solution was prepared in triplicate and kept under magnetic stirring of 125 rpm, in a shaker (GFL—model 1083), thermostatted at 25 ± 1 °C, in the time interval from 6 to 120 min [29].

The cachaça used in this study had a pH value of 4.0, which was maintained to simulate chitosan as an adsorbent in the current condition of the proposed application. 

The amount of copper adsorbed per unit mass of chitosan at equilibrium (Qe) (mg g^−1^) and removal efficiency (%R) [30] were calculated from Equations (9) and (10), respectively:

(9) $${\mathrm{Qe}=(C}_{0}{-\;C}_{e})V/M$$

(10) $${\%R=(C}_{0}{-\;C}_{t})/C_{0}\times 100\;$$

where C_0_ is the initial copper concentration in cachaça (mg L^−1^); C_e_ is the equilibrium of copper concentration (mg L^−1^); M is the mass of the adsorbent (g); V (L) is the volume of the cachaça solution, and C_t_ is the copper concentration at time *t*.

### 2.7. Kinetic Modeling

Four kinetic models were used to explain the mechanism of adsorption of copper by chitosan: Pseudo-first-order, Pseudo-second-order, Elovich, and Intra-particle Diffusion. 

Equation (11) shows the Pseudo-first-order kinetic model [31].

(11) $${\mathrm{dQ}}_{t}/{\mathrm{dt}=K}_{1}\left(Q_{e}\;{-\;Q}_{t}\right)$$

where Q_e_ and Q_t_ (mg g^−1^) are the amounts of copper ions adsorbed by chitosan at equilibrium and at time t (min), respectively; K_1_ (min^−1^) is the adsorption rate constant of the Pseudo-first-order model. The Pseudo-first-order model is verified from the graph slope (Q_e_ − Q_t_) as a function of t [32].

Equation (12) shows the Pseudo-second order kinetic model [33].

(12) $${\mathrm{dQ}}_{t}/{\mathrm{dt}=k}_{2}{\;(Q}_{e}{-\;Q}_{t}{\;)}^{2}$$

where Q_e_ and Q_t_ (mg g^−1^) are the numbers of copper ions adsorbed by chitosan at equilibrium and at time t (min), respectively; k_2_ is a proportionality constant of the Pseudo-second-order kinetic model (g mg^−1^ min^−1^). The applicability of the Pseudo-second-order model is observed from the graph of (t/Q_t_) against t [32]. 

The Equation (13) shows the Elovich equation. This equation covered a wide range of slow adsorption rates [34].

(13) $$d_{\mathrm{at}}/d_{t}={\;\alpha }_{e}-{\;\beta }_{\mathrm{qt}}$$

where α (mg g^−1^ min^−1^) is the initial adsorption rate, β (g mg^−1^) is the desorption constant, and q_t_ (mg^−1^) is the amount adsorbed at time t (min).

Another mathematical model used was the Intra-particle Diffusion [34] presented in Equation (14). This equation aims to identify a possible intra-particle diffusion mechanism as a limiting step.

(14) $$q_{t}=k_{p\;}t^{1/2}+\;C$$

where k_p_ (mg g^−1^ min ^1/2^) is the intra-particle diffusion rate constant; C (mg g^−1^) is the constant related to the resistance to diffusion, and q_t_ (mg g^−1^) is the amount adsorbed at time t (min).

## 3. Results and Discussion

### 3.1. Degree of Deacetylation

**Figure 1:**
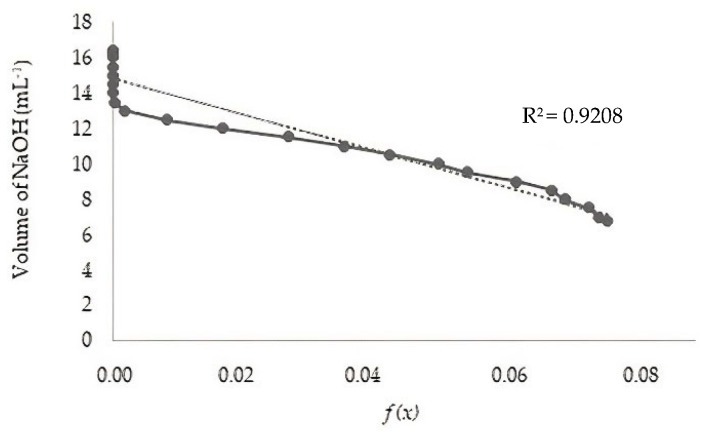
Linear titration curve for calculating the degree of deacetylation.

The *Q_t_* obtained showed an off-white appearance. The potentiometric titration and FTIR methods determined the DD. In the potentiometric titration method, the linear titration curve was obtained by plotting an f(x) graph as a function of the corresponding volume of NaOH solution (Figure 1).

The degrees of deacetylation determined by potentiometric titration and FTIR were 88.9 and 86.9%, respectively. These percentages show the two methodologies as suitable for this purpose. DD results found in the literature range from 50% to 95% [35,36]. Compared to the reaction time by microwave and conventional methods, the authors confirmed the reduction in reaction time by microwave irradiation, which allowed for energy savings [23].

Deacetylation by microwave radiation is an unconventional technology that has shown positive technological perspectives and bio-waste valorization, mainly due to advantages such as saving time and energy, efficiency, and sustainability [37]. The microwave heating mechanism is characterized by an ionic conduction and dipole rotation, resulting in rapid heating and, consequently, an improvement in the extraction of compounds [21,38].

The deacetylation yield in this study was 71.7%. This result is in agreement with the studies found in the literature [21]. As in this study, these authors also observed that the chitosan yield increased with the decrease in the size of the chitin particles and the increase in the concentration in the NaOH solution used for deacetylation (preliminary data not shown). Thus, the size of the chitin particles and the chitin-solvent ratio are factors related to extraction efficiency.

### 3.2. Molecular Weight of Chitosan

**Figure 2:**
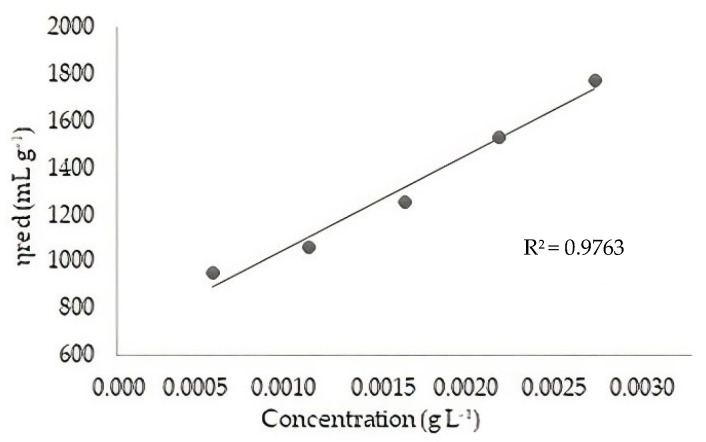
*Q_t_* Chitosan reduced viscosity curve.

The determination of the intrinsic viscosity of *Q_t_* was performed by linear regression from the values of reduced viscosity and the concentration of the chitosan solution (Figure 2).

The estimated molecular weight for *Q_t_* was 162.96 kDa. This value was calculated using the Mark–Houwink–Sakurada equation, with no standard classification range for molecular weight. However, several authors understand that low molecular weight chitosans are <50 kDa, average weight between 50–150 kDa, and high molecular weight >150 kDa. Following this criterion, the *Q_t_* obtained in this study was classified as high molecular weight [39]. The higher the molecular weight, the more entire segments the molecular chain has, increasing the viscosity of chitosan. Some studies show that the chitosan produced by the microwave technique has a higher molecular weight than the conventional method [23]. 

### 3.3. Attenuated Total Reflectance Fourier Transform Infrared Spectroscopy (ATR-FTIR)

**Figure 3:**
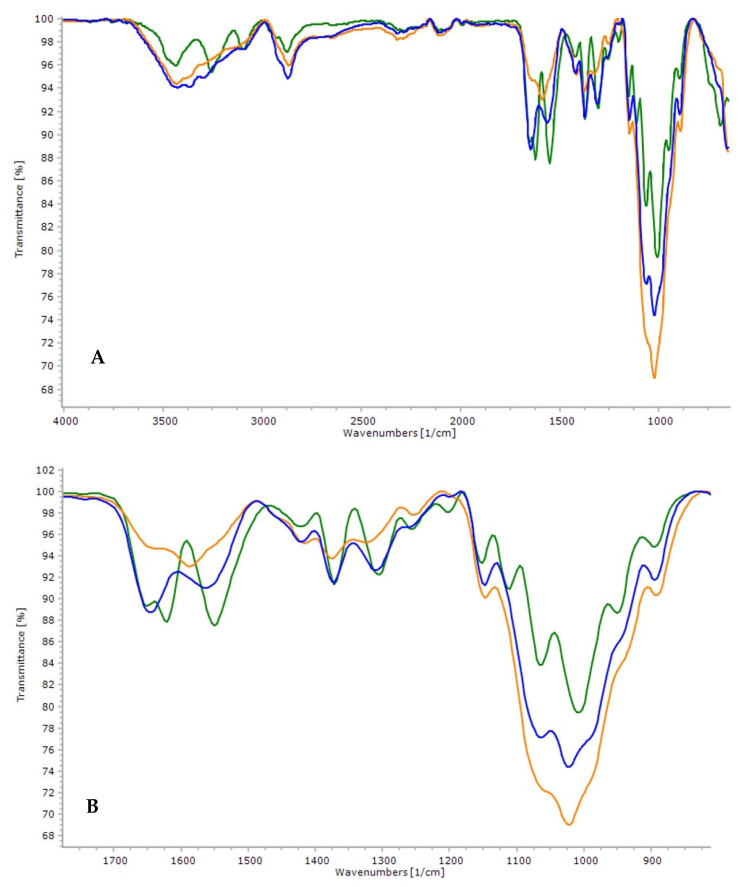
(**A**) Full infrared spectrum in the region of 4000 cm^−1^ to 600 cm^−1^ of *Q_i_* (**■**), *Q_t_* (**■**) and *Q_c_* (**■**); (**B**) Extended infrared spectrum in the of 1800 cm^−1^ to 800 cm^−1^.

The FTIR technique analyzed and identified the main functional groups present in chitosan structure and evidence of structural changes. Figure 3 illustrates the infrared spectra in the 4000 cm^−1^ to 600 cm^−1^ regions of the *Q_t_* sample, chitosan adsorbed with copper (II) (*Q_c_*) and chitin (*Q_i_*).

The bands in the 3430 cm^−1^ region, corresponding to the O–H elongation and intramolecular hydrogen bonds vibration, were observed in *Q_t_*, *Q_c_*, and *Q_i_*. The absorption band at 2850 cm^−1^ can be attributed to the elongation of the C–H group, typical bands characteristic of polysaccharides. The *N*-acetyl (acetamide) groups were confirmed by the bands around 1650 cm^−1^ (C=O The extension of amide I) and 1310 cm^−1^ (C–N extension of amide III), respectively. The bands from 1585 to 1550 cm^−1^ correspond to the N–H flexion of amines and secondary amides. The symmetrical deformations of CH_2_ and CH_3_ were confirmed by bands around 1420 and 1370 cm^−1^, respectively. The absorption band at 1145 cm^−1^ can be attributed to the elongation of C–O–C glycosidic bonds. The bands at 1060 and 1023 cm^−1^ correspond to the C–O elongation. The signal at 1255 cm^−1^ was assigned as C–O flexion vibrations and the signal at 895 cm^−1^ corresponds to the deviation of the CH out of the plane of the monosaccharide ring. The bands described are like those found in the literature [40,41]. 

In the spectrum obtained for *Q_i_*, in comparison to *Q_t_*, it is possible to notice that the band attributed to the functional group O–H that appeared at 3430 cm^−1^ was shifted to 3427 cm^−1^. When comparing *Q_i_*, *Q_t_*, and *Q_c_* in this same range, lower intensity of the band in *Q_i_* and higher for *Q_t_* and *Q_c_* are observed and, this may occur because nitrogen is less available for *Q_i_* (amides) hydrogen bonds than *Q_t_* and *Q_c_* (amines). The lower intensity at 1650 cm^−1^ for *Q_c_* suggests an inhibitory effect of copper (II) on the acetamide group (elongation C = O of amide I). The bands between 1585 and 1550 cm^−1^, related to amines flexion to secondary amides, have less intensity for *Q_c_* (1585 cm^−1^) and higher for *Q_i_* (1550 cm^−1^); this result corroborates the effect observed at 1650 cm^−1^ and confirms the more significant acetamide groups in chitin. A lower intensity value was observed for *Q_c_* at 1370 cm^−1^, which corresponds to the symmetrical deformations of CH_3_. It is possible to observe in the range between 1150 cm^−1^ and 894 cm^−1^ that copper (II) enhances the vibrational C–O–C interactions of glycosidic bonds (1150 cm^−1^). Axial and angular deformations of C–O bonds (1060 cm^−1^ and 1023 cm^−1^) are also enhanced by copper (II). The structural changes that influence the band’s displacements and their differences in intensities can be attributed to the interactions between chitosan and copper (II) through the groups of hydroxides, mainly of the amine groups present in the biopolymer [42].

### 3.4. Scanning Electron Microscopy (SEM)

**Figure 4:**
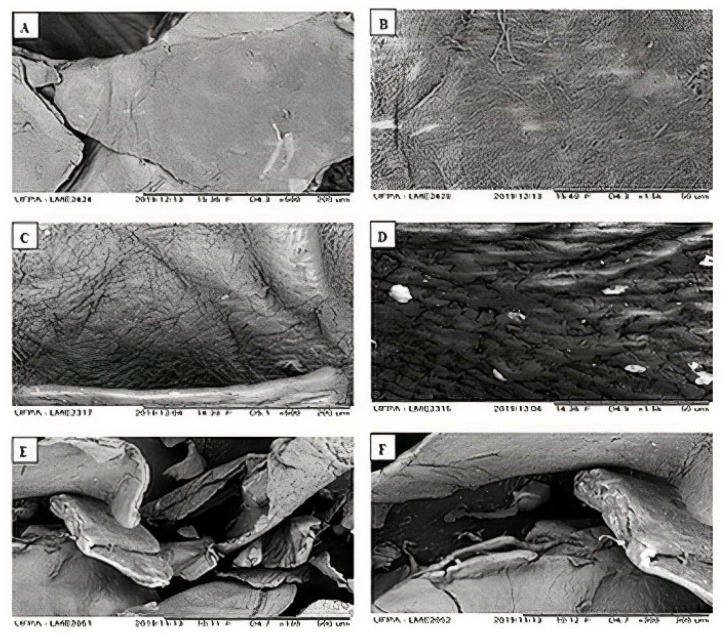
ERE photomicrographs magnified at 500 and 1500 times for *Q_i_* (**A**,**B**) and *Q_t_* (**C**,**D**). Magnified 180 and 300 times for *Q_c_* (**E**,**F**).

The cross-sectional sample of the backscattered electron imaging (BEI) were obtained by SEM (Figure 4):

It was possible to observe that the deacetylation reactions promoted structural changes. Photomicrographs (a) and (b) of *Q_i_* show a uniform and firm structure with organized fibers, characteristic of chitin particles. The structure of *Q_t_* (c) and (d), in turn, has a rougher surface, consisting of geometrically irregular particles, smaller than in the structure of *Q_i_*. Similar characteristics have been found in other studies [23,43]. Changes in the degree of deacetylation and the molecular weight in the polymer structure are directly related to the use of chitosan in specific applications, such as bio-adsorbent [44]. In the photomicrographs (e) and (f) of *Q_c_*, a surface with a more intense brightness is observed, characterized by that of Cu (II) in the structure of chitosan. The brightness intensity in BEI imaging is proportional to elements with a higher atomic number.

### 3.5. Energy Dispersion Spectroscopy (EDS)

**Figure 5:**
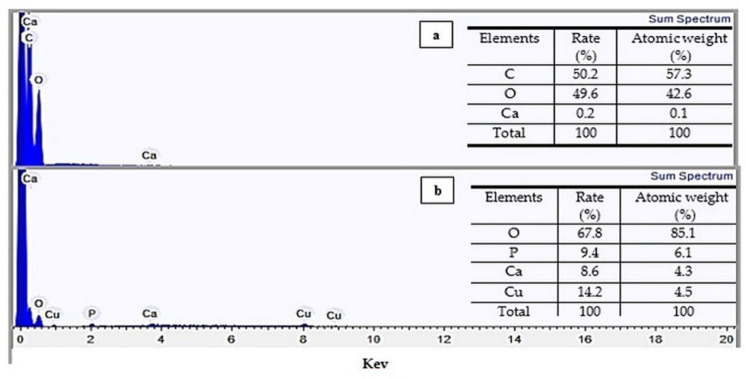
EDS spectra for *Q_t_* (**a**), *Q_c_* (**b**) and percentage of the elements found.

Chemical analyzes by EDS were performed to identify the elemental composition of chitosan. The spectrograms and their respective elementary percentages of *Q_t_* and *Q_c_* are shown in Figure 5.

The *Q_t_* presented C and O in greater quantity in elementary composition. A complex network of chitin and protein forms the structure of the crustaceans, where calcium carbonate deposits form the rigid shell. Thus, elements such as Ca and P can be found as residues from the demineralization and deproteination steps [45]. In the EDS spectra of *Q_c_*, the Cu (II) peak can be observed, which had a rate of adsorption of 14.2%; this represented a 71-fold increase over chitosan before adsorption and confirmed the presence of the metal ion in the biopolymer structure after the adsorption kinetics. 

### 3.6. Differentiation and Crystallinity by XRD

**Figure 6:**
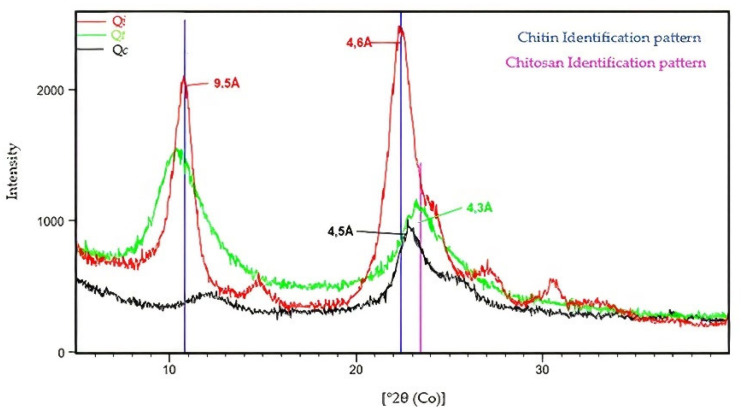
Comparison of the XRD standards for Q*i* (**■**), Q*_t_* (**■**) and Q*c* (**■**).

The XRD patterns were based on the 2θ position of the characteristic peaks of *Q_i_* (9.5° and 22.4°), *Q_t_* (9.1 and 23.5°) and *Q_c_* (22.9°), shown in Figure 6.

The diffraction profile of *Q_i_* is better defined due to its greater intensity and smaller amplitude. The XRD pattern of the *Q_t_* shows broad and medium intensity peaks, while the *Q_c_* shows a decrease in intensity, narrowing, and displacement of the peak from 23.5° to 22.9°. This displacement results from structural changes in *Q_t_* after Cu (II) adsorption. The changes can also be confirmed by increasing the distance *d*_hkl_ from 4.3 Å to 4.5 Å in the structure’s atomic plane (031). This crystalline variation occurs because of Cu (II) interactions in the biopolymer’s structure, forming a metal-chitosan complex. As shown in other research, the analyses showed that *Q_i_*’s atomic structure presents a better crystalline ordering about *Q_t_* and *Q_c_* [23].

**Figure 7:**
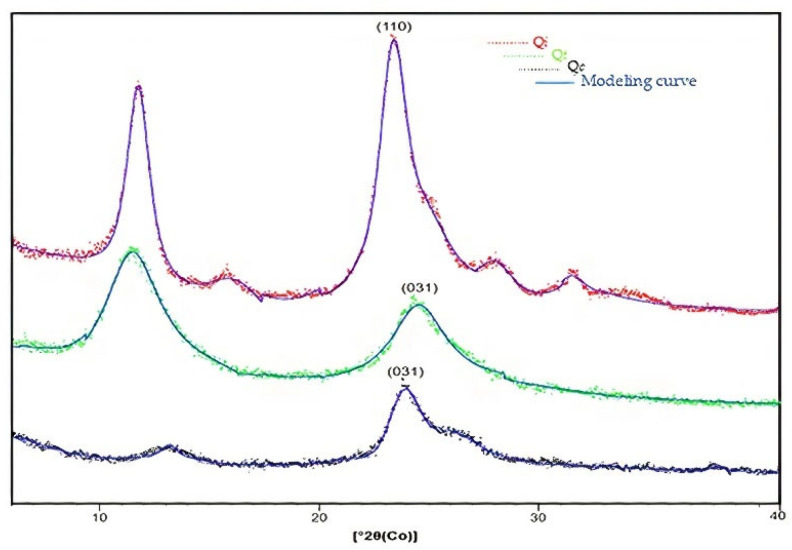
XRD standards modeled after the pseudoVoigt and Pearson VII functions in Highscore Plus.

For calculating the relative crystallinity of the compounds, the diffractograms were modeled linearly and exponentially through the Fit Profile application of the Highscore Plus software (Malvern Panalytical, Malvern, UK). This application combines the mathematical functions pseudoVoigt and Pearson VII generating a peak modeling curve (Figure 7). 

The values of the 2θ positions of the initial intensity base (I_0_) and the maximum intensity (I_max_) of the characteristic peak curve are reflected in Table 1 and Equation (7) [28]. Calculations show that *Q_i_* has more excellent crystallinity (84.9%) compared to *Q_t_* (54.2%) and *Q_c_* (59.2%). The crystalline differentiation between chitin and chitosan can be attributed to the breakdown of hydrogen interactions between the most electronegative groups of the material (–OH, NH_2_, and –C=O), which occurred during the deacetylation reaction.

The crystallinity of the biopolymer is a characteristic that can favor the accessibility of the metal ions to the adsorption sites; this is important because not all the amine groups (−NH_2_) present in the chitosan structure are available for the capture of metals. Some of these groups are involved in intra- or intermolecular hydrogen bonds. The degree of deacetylation is the characteristic that controls the fraction of amine groups available to interact with metal ions. The amine groups in chitosan are much more reactive than the acetyl groups in chitin [46].

In this study, in agreement with other authors, it is suggested that the interaction between the Cu (II) ion and the available electron pair of the amine group was responsible for the adsorption of the metal. The results observed in X-ray diffraction showed crystalline variations in the metal-chitosan complex. These variations suggest that crystalline phases have formed in the complex. A study investigated complexes of metal ions with chitosan using the X-ray diffraction technique. These authors observed that the complexes presented sharper X-ray diffraction bands than the chitosan bands, indicating the formation of crystalline phases in the chitosan induced by metal complexation [47].

### 3.7. Adsorption Kinetics

#### Kinetic Profiles

**Figure 8:**
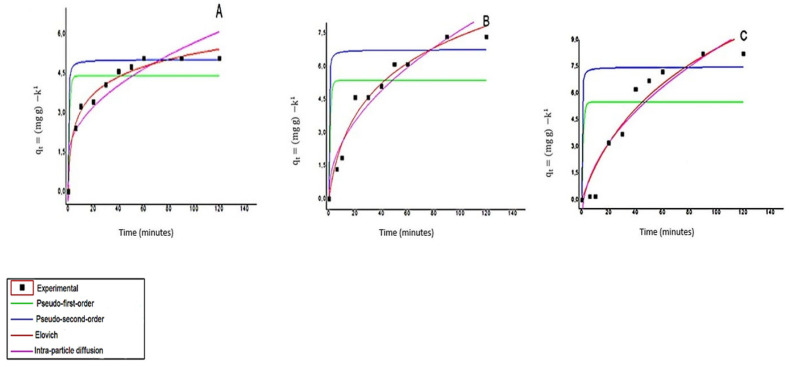
Kinetic profiles of Cu (II) adsorption in cachaça with a concentration of 50 mg L^−1^ for different values of *Q_t_* mass: 6 mg mL^−1^ (**A**); 4 mg ml^−1^ (**B**) and 2 mg ml^−1^ (**C**).

Figure 8A–C show the kinetic profiles of Cu (II) adsorption in different amounts of *Q_t_* mass (6 mg, 4 mg, and 2 mg per mL of cachaça), respectively. A rapid increase in the adsorption rate can be observed in kinetic profiles for all mass values in the first 20 min and a slower increase close to equilibrium. Agitation may have positively influenced reaching equilibrium due to increasing the probability of Cu (II) ions binding to active chitosan sites.

A more significant number of sites available for adsorption and subsequent metal saturation on the surface of the adsorbent justifies this behavior. The same behavior was found in studies comparing copper adsorption in materials based on chitin, chitosan, and modified chitosan [30]. 

Four linear kinetic models evaluated the mechanism that controls adsorption. Table 2 shows the kinetic parameters for Cu (II) adsorption for three different masses of chitosan.

Elovich’s kinetic model showed a better fit with the experimental data of chitosan among the models presented. In a complementary way, the values of the correlation coefficients show strong evidence that the chemisorption mechanism (chemical reaction) controls the ionic adsorption kinetics of copper by chitosan in cachaça, and this occurs because of the coordination and reaction between the solute and the amine and hydroxy groups in chitosan. The mechanism of chemosorption has also been found in other studies [48] in equilibrium and kinetics studies on the adsorption of surfactant, organic acids, and dyes for natural biopolymers. 

The equilibrium of copper adsorption in cachaça was reached in 60 min for the condition of 6 mg mL^−1^. This condition allowed the removal of 60.8% copper (II), while for the conditions of 4 mg mL^−1^ and 2 mg mL^−1^ the equilibrium was reached in 90 min and the removal was 58.8% and 38.8%, respectively. The rate of adsorption of copper removal achieved in this study is below some results found in the literature for the removal of copper ions, using other adsorbents in cachaça, such as modified silica-titania (90%) [2], bentonite (88%) [49] and activated carbon (99%) [10]. It was important to note that the chitosan obtained (*Q_t_*) and used in this adsorption study has not been chemically modified. The natural structure was maintained to reduce costs and establish technological viability. The original pH value of cachaça was maintained to simulate the natural conditions of the use of chitosan. The pH of 4.0 may have influenced the availability of adsorption sites. Thus, more protons will be available at low pH to protonate the amine groups and form –1NH^3+^ groups, reducing the number of binding sites for Cu (II) adsorption.

The increase in the adsorbent concentration improved the removal efficiency of copper ions and, this can be attributed to an excess of adsorption site (amine groups, hydroxyl), available on the surface of chitosan, and binding to metallic ions. Other studies have shown a similar effect. [30].

### 3.8. Quantification of Copper by Microwave-Induced Plasma Optical Emission Spectrometry (MIP OES)

Table 3 shows the levels of Cu (II) in the three samples (cachaça, cachaça doped with copper (50 mg L^−1^) and cachaça after adsorption) analyzed using the MIP OES technique.

The theoretical concentration of 50 mg L^−1^ was established, corresponding to 10 times the limit of Brazilian legislation and 25 times the international legislation. The results show that after the adsorption, the final concentration of Cu (II) in cachaça was 7.7 mg L^−1^ and, this represented an 84% reduction in the initial concentration. Thus, it was observed through the established experimental conditions that the chitosan obtained in this study showed a high capacity to adsorb copper in cachaça, even without modification in its structure.

Several studies use the MIP OES technique due to its better feasibility and lower cost in determining heavy metals when compared to other techniques such as Flame Atomic Absorption Spectrometry [50]. In this study, MIP OES was used as a confirmatory technique of greater accuracy to determine the concentration of Cu (II) in cachaça after adsorption. Studies with the MIP OES technique in different beverages can be found, for example, in multielement determination in instant soups [51]; in standard dilution analysis of beverages [52]; in the determination of major and minor elements in Mexican red wines [53]. In general, the use of MIP OES for food analysis is attractive due to its efficiency and low cost.

## 4. Conclusions

In conclusion, from the experimental evidence obtained in this work, we can say that chitosan, obtained by microwave irradiation, presented satisfactory properties in the main characteristics observed. The degree of deacetylation, molecular weight, morphology, and crystallinity confirmed the effectiveness of deacetylation. In the biopolymer application, the Cu (II) adsorption capacity results show that maximum adsorption removal was higher in the kinetic condition with the highest mass of chitosan within the range studied. However, some authors do not always observe this phenomenon in other conditions. An excess of adsorption sites is available on the surface of the biopolymer. The chemisorption mechanism best explained the adsorption kinetics. The 84% reduction of Cu (II) in cachaça shows that chitosan can remove this ion. This study shows the application potential of natural chitosan (without chemical modification) from shrimp shells as a bioadsorbent in the pot still cachaça. Furthermore, the microwave deacetylation process of chitin is considered a clean technology. This opens a real possibility for the technique to be improved in future applications.

## Figures and Tables

**Table 1:** Results obtained to the 2θ position: Distance *d* from the atomic planes and intensity of the characteristic peaks.

Sample	Characteristic Peak	*d*_hkl_ * (Å)	I_max_ **	I_0_ ***	Relative Crystallinity (%)
Chitin	2θ = 22.4° (*h k l* = 110)	4.6	2451	368	84.9
Chitosan	2θ = 23.5° (*h k l* = 031)	4.3	1086	479	54.2
Chitosan adsorbed with Cu (II)	2θ = 22.9° (*h k l* = 031)	4.5	924	377	59.2

*d*_hkl_ *: distance d from atomic planes; I_max_ **: maximum intensity; I_0_ ***: initial intensity of the values of the 2θ positions of the characteristic peak curve.

**Table 2:** Parameters related to the analyzed kinetic models.

Models	Parameters	6 mg mL^−1^	4 mg mL^−1^	2 mg mL^−1^
Pseudo-first-order	K_1_ (min^−1^)	1	1	1
	Q_e_ (mg g^−1^)	4.3883	5.3617	5.4830
	R^2^	0.6192	0.2639	0.0744
Pseudo-second-order	K_2_ (g mg^−1^ min^−1^)	1	1	1
	Q_e_ (mg g^−1^)	5.0079	6.7516	7.4483
	R^2^	0.4036	−21485	−50261
Elovich	α (mg g^−1^ min^−1^)	2.1716	0.4205	0.2215
	β (g mg ^−1^)	1.0359	0.3857	0.1985
	R^2^	0.9780	0.9665	0.9179
Intra-particle-diffusion	K_p_ (mg g^−1^ min^−1/2^)	0.4413	0.7449	0.9591
	C (mg g^−1^)	1.2383	0.1832	−1.1005
	R^2^	0.8139	0.9291	0.8858

**Table 3:** Average copper levels (mg L^−1^) in the cachaça samples analyzed and their respective standard deviations (*n* = 3).

Sample	Copper (372.395) *^,^ª
Cachaça	3.4 ± 0.003
Cachaça with copper	48.4 ± 0.52
Cachaça after adsorption	7.7 ± 0.11

* Wave-length; ª Atomic line.

## Data Availability

The data presented in this study are available on request from the corresponding author.
